# High-Sensitivity C-Reactive Protein and Carotid Intima Media Thickness as Markers of Subclinical Inflammation and Atherosclerosis in Pediatric Patients with Hypercholesterolemia

**DOI:** 10.3390/molecules25215118

**Published:** 2020-11-04

**Authors:** Lana Blinc, Matej Mlinaric, Tadej Battelino, Urh Groselj

**Affiliations:** 1 Faculty of Medicine, University of Ljubljana, Vrazov trg 2, 1000 Ljubljana, Slovenia; lana.blinc@mac.com (L.B.); tadej.battelino@mf.uni-lj.si (T.B.); 2 University Children’s Hospital, University Medical Center Ljubljana, Bohoriceva ulica 20, 1000 Ljubljana, Slovenia; 19matej91@gmail.com

**Keywords:** familial hypercholesterolemia, polygenic hypercholesterolemia, inflammation, atherosclerosis, carotid intima media thickness, cIMT, C-reactive protein, hsCRP

## Abstract

Hypercholesterolemia is a major cause of atherosclerosis development and premature cardiovascular disease (CVD). It leads to inflammation, which further accelerates atherosclerosis progression. Familial hypercholesterolemia (FH) is an autosomal dominant disorder characterized by elevated serum LDL-c from birth, due to a disease-causing variant in one of the causative genes (*LDLR*, *APOB*, *PCSK9*). In polygenic hypercholesterolemia (PH), the disease-causing genetic variant is absent; it is likely the cumulative result of multiple single nucleotide polymorphisms in LDL metabolism-related genes and other factors, such as lifestyle and environment. In high risk groups, such as patients with FH, an effective primary prevention of CVD must begin in childhood. High-sensitivity C-reactive protein (hsCRP) and carotid intima media thickness (cIMT) are two potential minimally invasive correlates of inflammation and subclinical atherosclerosis progression. hsCRP and cIMT have been shown to be significantly increased in patients with FH and PH relative to healthy controls, with some studies yielding conflicting results. In this review, we aim to summarize current knowledge and recent findings regarding the applicability of hsCRP and cIMT as markers of low-grade inflammation and subclinical atherosclerosis, focusing especially on children and adolescents with hypercholesterolemia.

## 1. Introduction

Hypercholesterolemia is a major cause for early atherosclerosis development and premature cardiovascular disease (CVD), which are the main global causes of morbidity and mortality [1]. Hypercholesterolemia in childhood, adolescence, and young adulthood is associated with atherosclerosis and its complications later in life [2,3]. In most cases, hypercholesterolemia is of multifactorial (or polygenic) etiology, while in other cases it is monogenic, mostly heterozygous familial hypercholesterolemia (FH) [4,5]. FH is an autosomal dominant disorder characterized by elevated serum LDL-c from birth on due to a disease-causing variant in one of the causative genes (*LDLR*, *APOB*, *PCSK9*) [5]. In polygenic hypercholesterolemia (PH), the disease-causing genetic variant is absent; it is likely the cumulative result of multiple single nucleotide polymorphisms in LDL metabolism-related genes and other factors, such as lifestyle and environment [4,6]. Permanently elevated low-density lipoprotein (LDL) cholesterol levels in plasma oxidize and begin to accumulate in the walls of the arteries, triggering inflammation [7]. This results in vascular injury and plaque formation [7,8]. In groups at high risk, such as patients with FH, an effective primary prevention of CVD must begin in childhood [6,8].

It is shown that the cholesterol crystals in the vascular wall induce the inflammatory response with the activation of the NOD- (nucleotide-binding oligomerization domain-like receptor), LRR- (leucine-rich repeat), and pyrin domain-containing protein 3 (NLRP3) inflammasome, which induces an inflammatory cascade via caspase-1, which in turn activates the cytokine pathway. This might be one of the crucial steps in initiating the process of atherosclerosis. Deposition of cholesterol to the vascular wall might thus cause (and not result from) the inflammatory process [9]. High-sensitivity C-reactive protein (hsCRP) and carotid intima media thickness (cIMT) are two potential minimally invasive correlates of inflammation and subclinical atherosclerosis progression [10,11,12,13]. hsCRP can be shown to be significantly increased in patients with both FH and PH relative to healthy controls and has been shown to decrease after the implementation of healthy lifestyle habits in patients with stable CVD [14,15,16]. Similarly, cIMT is shown to be greater in patients with FH as compared to their siblings, and the rate of cIMT progression is decreased in children with FH receiving statin therapy [17]. While diagnostic and treatment protocols have been established, tools for risk stratification would significantly aid in patient management according to the concept of precision medicine [18].

In the present review, we aim to examine the recent findings regarding two potential biomarkers of atherosclerosis progression and inflammation—hsCRP and cIMT—also focusing on their role in the pediatric population.

## 2. Familial and Polygenic Hypercholesterolemia—Outlining Prevalence, Pathophysiology, Diagnosis, and Treatment

FH represents one of the most common genetic global causes for premature CVD, with an estimated prevalence of 1/311 in the general population [19]. PH denotes the proposed etiology of hypercholesterolemia in patients who meet clinical, but not genetic criteria for FH, as no known causal mutation can be identified. The estimated proportion of patients with PH in those with clinically definite FH is about 30%, and even greater (up to 70%) in those with clinically possible FH [6,20].

Several mutations are known to cause FH. The vast majority (up to 85%) are found in the low-density lipoprotein receptor gene (*LDLR*). Over 1700 LDLR mutations have been described, with 79% probably presenting as a hypercholesterolemic phenotype [8,21]. They may cause aberrant protein synthesis, impaired post-translational modification, flawed interaction between LDLR and its ligand, apolipoprotein B100 (ApoB100) on the surface of low density lipoprotein cholesterol (LDL-c) particles, reduced endocytosis of the LDLR-LDL-c complex, or derailment of the intracellular “recycling” of LDLR (wherein the receptor is separated from its ligand and returned to the surface of the cell) [21]. Other genes in which causal mutations have been identified include ApoB100, proprotein convertase subtilisin/kexin type 9 gene (*PCSK9*), and lipoprotein receptor adaptor protein 1 gene (*LDLRAP1*). The majority of FH patients are heterozygotes, and the disease is inherited in an autosomal dominant pattern. In homozygotes, the mode of inheritance is either autosomal codominant or autosomal recessive (the latter in the case of *LDLRAP1* mutations) [8,21,22]. PH is thought to be the cumulative result of single nucleotide polymorphisms (SNPs) in several genes affecting LDL-c concentrations, including *LDLR* and *ApoB* [23]. In patients who meet clinical criteria for FH but do not have identifiable causal mutations or SNPs, it is assumed that the clinical presentation arises from mutations in gene sections beyond the scope of current analytical methods, epigenetic modifications, mutations in unknown genes, or environmental factors [24].

FH and PH result in impaired LDL-c metabolism, which manifests as high blood concentrations of LDL-c and total cholesterol. This in turn contributes to accelerated atherosclerosis through oxidation, endothelial dysfunction and inflammation, and ultimately results in premature CVD, most commonly manifesting as acute myocardial infarctions and angina pectoris, as well as aortic valvular stenosis and xanthomata at tendinous insertions, large joints, and medial canthi in more severe cases [8,22,25,26]. The average LDL-c blood concentration in the untreated heterozygous form of FH is 5.7 mmol/L and 12.9 mmol/L in the untreated homozygous form [25,26]. In PH, the LDL-c concentrations are similar as in the heterozygous form of FH, though diagnosis of PH is more likely at lower LDL-c concentrations. This demonstrates the incremental contributions of individual SNPs to increasing LDL-c concentrations, and their presence may affect the range of LDL-c concentrations in relatives of patients with confirmed FH [23,27].

There are several diagnostic criteria for FH; the most frequently used (e.g., Simon Broome criteria and the Dutch Lipid Network Criteria) combine the clinical presentation, family history, and genetic analysis and classify patients according to the likelihood of having FH [23,25]. For PH, polygenic risk scores, developed by defining high-risk SNPs through genome wide association studies, enable researchers to evaluate patients’ risk of CVD and related complications, but are not yet available for clinical use [23]. Implementation of population screening is justified by the following arguments. (1) FH is a common and decidedly underdiagnosed condition with a high probability of severe complications if left untreated; (2) childhood represents a latent period in disease progression, during which treatment initiation has the largest downstream effect; (3) an appropriate screening method is available and applicable in the primary care setting. [8,19,28]. Statins are the therapy of choice in adult and pediatric populations with FH [8,26,28]. Despite being effective and well tolerated, they may be insufficient for the recommended decrease in LDL-c concentrations, and can be supplemented with ezetimibe, bile acid sequestrants, lipoprotein apheresis, and PCSK9 inhibitors [8,21,28,29,30]. In PH, treatment may be less aggressive due to the relatively lower LDL-c concentrations and may be carried out in the general practice setting [23].

## 3. High-Sensitivity C-Reactive Protein as a Marker of Subclinical Inflammation in Atherosclerosis

### 3.1. The Role of C-Reactive Protein in Inflammation and Atherosclerosis

C-reactive protein (CRP) is a well-known human acute phase protein and is frequently assessed by clinicians to monitor infections, trauma, response to antibiotics, and activity of autoimmune conditions. It is a pentameric molecule formed by five identical monomers, and in the acute phase response, its plasma concentrations can increase over 1000-fold compared to baseline within a few hours [12,13]. It is synthesized in hepatocytes and some extrahepatic tissues, including vascular smooth muscle, atherosclerotic plaques, and intracardiac tissues, in response to several cytokines, including interleukin-6 and interleukin-1 [12,13,31]. Its rate of synthesis depends on the intensity of the pathology driving it—its concentration doubles every 8 h and reaches its peak within 36 to 50 h. Its half-life is around 19 h, and elimination is mainly mediated by the liver. Basal concentration appears to depend on age, sex, hormonal condition, smoking status, obesity, comorbidities, genetic polymorphisms, and other factors; therefore, the average of two measurements should be used. In healthy individuals, blood concentrations usually do not exceed 10 mg/L. Values lesser than 0.8 mg/L are difficult to accurately assess by regular methods, which is why high-sensitivity assays (with the subsequent addition of the “high-sensitivity” prefix to the parameter, i.e., high-sensitivity CRP, hsCRP) have evolved for use in the experimental setting, and increasingly in the clinical setting [13].

Two prominent functions of CRP are activation of the classical complement pathway via C1q binding, and binding to human immunoglobulin Fcγ receptors with subsequent opsonization of ligands for macrophages [12]. It is found, together with activated complement fragments and activated macrophages, in human atherosclerotic lesions, where it may contribute to chronic inflammation. As it binds to various forms of LDL-c and is taken up by macrophages together with LDL-c via Fcγ-dependent and Fcγ-independent pathways, it may contribute to foam cell formation in atherogenesis. In vitro studies also suggest its role in regulating complement activation in atherogenesis [12].

A number of other in vitro effects on vascular cells have been suggested (including increased expression of adhesion molecules and decay accelerating factor (DAF) on endothelial cells, secretion of chemokines, etc.), but these results must be interpreted with caution, as the effects may be due to contamination of commercially available CRP (cCRP) [12] Specifically, van den Berg et al. and Taylor et al. demonstrated the inability to reproduce vasorelaxation or increased expression of DAF by using their own recombinant CRP, as well as CRP purified from human ascites, and found that the effects stemmed from the presence of azide in cCRP [32,33]. In other studies, using azide-free CRP, no increased adhesion of monocytes to endothelial cell cultures was observed, and minimal chemokine secretion was observed. Further, they report that azide in cCRP also induces change in endothelial cell culture morphology, inhibition of proliferation and viability, reduces the expression of endothelial nitric oxide synthase (eNOS), and that lipopolysaccharide (LPS) and azide, but not CRP, increase expression of certain adhesion molecules. Additionally, they found that azide also decreases secretion of von Willebrand’s factor, and LPS mediates the increased secretion of interleukin-8 and monocyte chemoattractant protein-1 [33]. Interestingly, Devaraj et al. observed that the addition of CRP purified from human ascites to endothelial cell culture decreased eNOS activity and increased monocyte adhesion [34]. They examined this effect in vivo using rats and demonstrated impaired vasodilation [34]; however, the use of rats as animal models for experiments relating to CRP is problematic, as they have very high basal CRP plasma levels and are thus a poor approximation of human physiology [12]. Bisoendial et al. demonstrated increased inflammation and coagulation following an injection of highly purified human recombinant CRP in seven healthy adult male volunteers [35,36]. Data from Mendelian randomization studies do not support the pathogenicity of CRP in atherosclerosis, but inferring causality from such studies may prove challenging—confounders include pleiotropy of CRP SNPs and their influence on other biomarkers associated with CVD, interactions between genes, etc. [37]. In summary, more research is needed to elucidate whether CRP causally contributes to atherosclerosis progression in humans.

### 3.2. High-Sensitivity C-Reactive Protein as a Predictor of Cardiovascular Disease

Several prospective studies have highlighted the association between basal CRP concentrations and future cardiovascular events in individuals with no CVD. In the Physicians’ Health study involving nearly fifteen-thousand healthy men, it was shown that higher basal hsCRP coincides with higher risk of stroke, myocardial infarction, and severe peripheral artery disease [13,37,38]. A subsequent study in a large sample of healthy postmenopausal women suggested that hsCRP may be a better prognostic factor for cardiovascular events than homocysteine or lipids [39]. The results of a subsequent meta-analysis by Kaptoge et al. show that hsCRP is a more accurate prognostic factor for CVD than increased arterial blood pressure or cholesterol concentration [40]. Still, the results of several other studies dispute this proposed prognostic value of hsCRP. The Heart Protection Study showed that baseline hsCRP does not modify vascular benefits of statin therapy and inferred that the relationship of hsCRP concentration and CVD risk may be explained by other risk factors [41]. Shah et al. conducted a systematic review of 31 prospective cohorts and found that, overall, CRP did not perform better than the Framingham risk equation, and that the improvement of risk stratification through the addition of CRP to established predictive models is small and inconsistent [42]. The systematic review by Schnell-Inderst et al. reports that, while the addition of hsCRP to traditional risk factors improves risk prediction, the clinical relevance and economic benefit of this addition remain unclear [43]. The 2016 European guidelines on cardiovascular disease prevention state that, while hsCRP has shown consistency across large prospective studies as a risk factor integrating multiple metabolic and low-grade inflammatory factors, its contribution to the existing methods of cardiovascular risk assessment is probably small. Consequently, they advise against routine assessment of circulating biomarkers (including hsCRP) for refinement of CVD risk assessment (recommendation class III level B), especially in patients with clearly high or low risk. They add that there is evidence of publication bias in the field of novel biomarkers of cardiovascular risk, leading to inflated estimates of strength of association and potential added value [44].

### 3.3. High-Sensitivity C-Reactive Protein in Pediatric Patients with Familial and Polygenic Hypercholesterolemia

Research relating hsCRP to patients with FH and PH is relatively sparse, especially for pediatric patients. van Wissen et al. showed greater hsCRP reduction in patients receiving 80 mg of atorvastatin than in patients receiving 40 mg of simvastatin and a correlation between hsCRP decrease and cIMT reduction [45]. Rahman et al. showed not only higher concentrations of hsCRP in FH patients (prior to statin initiation) relative to normolipemic control subjects, but also in the unaffected relatives of FH patients [14]. A review by Narverud et al. found 11 studies on CRP levels in children with FH. Five describe significantly higher CRP levels in children with FH relative to age-matched controls, but the rest found no significant difference. The authors concluded that CRP may not be an equally sensitive marker in children and in adults [46]. Additionally, Narverud et al. found no significant differences in CRP levels between a cohort of children with the severe, LDL receptor-negative form of FH, and a cohort of children with other forms of the disease. They also compared CRP levels relative to paternal or maternal inheritance of mutations and found no significant differences. They concluded that the primary driver of atherosclerosis in these patients is not the inheritance pattern, but rather the lifelong hypercholesterolemia burden conferred by causal mutations [47]. Ueland et al. found significantly higher levels of hsCRP in children with FH as compared with unaffected siblings, but their hsCRP levels were only 10% of the hsCRP levels observed in healthy adults in other studies. Furthermore, no correlation was observed between hsCRP and cIMT [48]. Calan et al. demonstrated increased levels of hsCRP, as well as adhesion molecules and homocysteine in a sample of PH patients relative to both patients with isolated hypertriglyceridemia and controls [15]. Guran et al. found significantly higher hsCRP levels in groups with risk factors for coronary heart disease (hypercholesterolemia, hypertension, obesity, low HDL cholesterol, and family history of coronary heart disease) compared to healthy children. A positive correlation between hsCRP and body mass index was observed, but no correlation between hsCRP and total cholesterol or LDL-c concentrations was found [49].

Other laboratory markers of atherosclerosis are also under scrutiny. One such example is microRNA. In the study by Martion et al., miR-33a and miR-33b, which regulate pathways controlling HDL cholesterol levels, TG, and insulin signaling, were found to be upregulated in children in FH, and a correlation between their levels, and the levels of CRP was also observed. However, as the sample was small, further studies are required. [50].

In a study by Christensen et al., monocytosis (with an observed shift toward higher numbers of circulating proinflammatory and non-classical monocytes) was found in children with FH. hsCRP correlated positively with proinflammatory monocytes and inversely with classical monocytes. Additionally, statistically significant differences were observed between the levels of hsCRP in children with FH relative to healthy controls, but this was not the main focus of the study [51].

Given the relative paucity of research and occasionally conflicting results, further studies are required to fully establish the utility of hsCRP as a marker of low-grade inflammation in children with FH and PH. At present, hsCRP is not recommended for refining CVD risk assessment in FH patients as per the 2016 European guidelines on cardiovascular disease prevention, as these patients are categorized as high- or very high-risk in the 2019 European Society for Cardiology/European Atherosclerosis society guidelines for dyslipidemia management. While PH patients are not specifically mentioned in the 2019 European Society for Cardiology/European Atherosclerosis society guidelines for dyslipidemia management, similar recommendations may be inferred from the 2016 European guidelines on cardiovascular disease prevention, given the caveats of hsCRP in refinement of CVD risk listed in Section 3.2 [44,52].

## 4. Carotid Intima-Media Thickness as a Measure of Subclinical Atherosclerosis

### 4.1. Defining Carotid Intima Media Thickness as a Parameter

Intima media thickness (IMT) is defined as the area of tissue beginning at the luminal edge of the artery and ending at the boundary between the media and adventitia, seen as parallel lines using B-mode ultrasound [53]. Pignoli et al. were the first to report in vitro results of their study investigating the thickness of the arterial wall with B-mode ultrasound [54]. They found that the distance between two parallel echogenic lines correlated closely with the IMT measured upon pathological examination of the tissue and concluded that the B-mode ultrasound was a useful modality for in vivo IMT measurements. Subsequently, the high reproducibility of B-mode ultrasound measurements of IMT was demonstrated. Uniform thickening of IMT has been shown to occur with increasing age, and all known major vascular risk factors contribute to the increase and progression of IMT thickening [10]. It is, however, important to note that at the individual level, the measurement is not strictly associated with risk of stroke, myocardial infarction and peripheral artery disease [53]. Indeed, IMT may not exclusively reflect early atherosclerosis—epidemiological studies have shown that non-atherosclerotic medial hypertrophy also occurs, and unified criteria are required to distinguish it from atherosclerotic changes, as the two phenomena exhibit differences in localization, natural history, risk factors and predictive value for cardiovascular complications [53]. The carotid IMT (cIMT) has become a frequently measured parameter in research and clinical practice, not least because of easy access due to its anatomical position, non-invasiveness of the procedure and its relatively low cost. It is a frequently used surrogate endpoint in clinical trials owing to the assumption that changes in cIMT correlate directly with changes in cardiovascular risk [10].

### 4.2. Applying Carotid Intima Media Thickness as a Tool for Cardiovascular Disease Risk Stratification at the Individual Level vs. as a Surrogate Endpoint in Clinical Trials

A meta-analysis by Liao et al. [55] questioned the benefit of using cIMT as a clinical tool for the purpose of individual patient risk stratification for CVD complications. While it acknowledges the relative consistency of cIMT in predicting vascular events among various cohorts, which is in agreement with several other meta-analyses [10,11,56], it sheds light on an important pitfall for individual clinical applicability—the lack of consistent normative values. The authors found that the heterogeneity between measurements in different cohorts was so vast that no clear reference values could be established. When discussing the reasons behind such large discrepancies, they state that the differences could not be explained by ultrasound protocols, ethnicity, or geographical factors, and list residual population differences; unaccountable aspects of geography/ethnicity; and the effects of individual ultrasound machines, probes, protocols, and technician influence as possible confounders [55]. The authors of the study acknowledge the conflicting findings between their own analysis and that of Engelen et al. [57], wherein the researchers estimated the age- and sex-specific cIMT reference intervals in a healthy population, and assessed the association between cardiovascular risk factors and cIMT reference intervals in order to enable comparison between patients at varying CVD risk levels and healthy individuals. Liao et al. suggest the much greater heterogeneity in cIMT measurement techniques and ethnicity in their own study as a possible explanation [55].

There appears to be a similar lack of agreement regarding cIMT normative values in pediatric cohorts as well. Several studies sought to determine the effect of various parameters (including age, sex, body mass index (BMI), blood pressure, lipid profile, and insulin resistance) on cIMT in healthy pediatric cohorts and reported conflicting results. Gooty et al. [58] reported no significant age difference in cIMT from childhood to adolescence, as measured in their cohort of 291 healthy children, aged 6 to 18. They noted a correlation between BMI and CVD risk score (calculated from the average of age appropriate standardized deviates of the primary components of metabolic syndrome), as well as an association between said parameters and cIMT. In contrast, Baroncini et al. [59] reported no correlation between cIMT and BMI, or between cIMT and sex in their cohort of 280 healthy children and adolescents but did note an increase in cIMT in children aged 11 to 15. Zanini et al. [60] observed higher values of cIMT in male adolescents, and a positive correlation between cIMT and pubertal development in their cohort of 61 healthy adolescents. Calabrò et al. [61] found a positive correlation between cIMT and age, height, systolic blood pressure and BMI—all only in boys within a cohort of 131 healthy children and adolescents aged 3 to 16. Similarly, Curcio et al. [62] noted statistically significant higher values of cIMT in boys over 15 years old in their cohort of 161 healthy individuals aged 4 to 28. Koçyiğit et al. [63] found no sex-related differences in cIMT irrespective of age, but noted a positive correlation between cIMT and age, as well as no correlation between cIMT and systolic blood pressure, in their cohort of 91 healthy children aged 7 to 15. The conflicting results may be explained by differences in cIMT measurement protocols, inherent variability between cohorts (e.g., differences in included age spans between studies, relatively small samples), and lack of longitudinal observation—all the studies listed are cross-sectional, allowing limited insight into individual progression of cIMT. While the Scientific Statement published by the American Heart Association in 2009 lists normative values for different pediatric age groups, the studies that provided the information also appear to have inconsistent results, as well as overlap in cIMT values between age-categories [64]. The statement from the Association of European Pediatric Cardiology published in 2014 (Dalla Pozza) [65] includes advice on patient selection, a proposition for a standard protocol of cIMT scanning and analysis, and supplies normative values from several studies; however, the values, age groups, and sample sizes vary. The authors emphasize the need for longitudinal data regarding the benefit of cIMT for estimating cardiovascular morbidity and mortality, and for elucidating which patient groups especially benefit from cIMT measurements.

A meta-analysis by Lorenz et al. [56] examined the association between cIMT progression and increases in risk of CVD in the general population. The researchers found no evidence of association between individual cIMT progression and risk of subsequent CVD, regardless of definition of cIMT, endpoint, or adjustment. These findings contrast with those from the study by Polak et al. [66], in which researchers found a significant positive correlation between yearly mean common cIMT progression and risk of stroke. Because Lorenz et al. included comparable multi-ethnic cohorts in their own analysis they reject an ethnic-dependent effect as the explanation and suggest that the findings of Polak et al. may be spurious [56]. Despite finding no association between individual cIMT progression and risk of subsequent CVD, Lorenz et al. confirm a statistically significant association between mean cIMT and subsequent clinical endpoints. They suggest several possible explanations for the first conclusion: (a) factors affecting progression estimates, including population heterogeneity; (b) reduced reliability of repeated cIMT measurements; (c) the confounding effect of potential plaque superimposition at sites of maximal cIMT increase, along with different respective associations between plaque presence or cIMT progression and clinical endpoints; (d) the Hawthorne effect, i.e., individual behavioral change due to participation in a longitudinal population study. The authors state that the latter is unlikely, as the effect in the general population is thought to be minimal, has not been substantiated for smoking cessation, and only a small number of included studies informed participants of their cIMT values [56].

Lorenz et al. also examined the predictive value of cIMT and its progression rate for CVD in individuals at high risk [67]. They list three ways in which they avoided duplicating data from their previous study: (1) selective analysis of individuals with high cardiovascular risk from the general population cohorts they previously examined, which might have improved the ratio between the hypothesized association and measurement error; (2) the addition of several new cohorts according to the modified inclusion criteria; and (3) the addition of a highest-risk cohort that was excluded from their previous analyses. As in the 2012 meta-analysis, they found no association between cIMT progression and future CVD event risk but demonstrated a significant association between average common cIMT and subsequent risk of CVD complications. The authors also found that, even in high-risk populations, annual cIMT was not a reproducible measurement—i.e., there was no correlation in cIMT change between the first–second scan, and the third–fourth scan. They propose measurement errors as a plausible explanation, as finding the exact same spot in consecutive ultrasounds several years apart may prove challenging. Non-atherosclerotic medial remodeling, along with atherosclerotic plaque superimposition, especially in patients with high CVD-event risk, may obfuscate the atherosclerotic non-plaque increase in cIMT and contribute to the lack of correlation demonstrated in the study. While the authors mention that they also analyzed cohorts where plaques were excluded from cIMT measurements, they state that no significant differences were found between the two sets of conclusions. They propose difficulty of differentiating between ultrasonic images of plaques and cIMT, also due to the lack of standardization process for plaque measurement and limited data, as a possible reason. They state that despite attempting to correct for antihypertensive and lipid lowering treatment, it is possible that complex interactions between lifestyle factors, treatment and cIMT may blur the association between CVD risk and cIMT progression. Finally, they emphasize the difference between the individual surrogacy of cIMT progression as a marker for CVD risk, addressed in the study, and the progression of cIMT as a surrogate endpoint in clinical trials. For the latter, they assert a lack of consensus and the need for further research [56,67].

To that end, Willeit et al. [68] very recently published a meta-analysis in which they address the adequacy of cIMT as a surrogate endpoint in clinical trials involving the adult population (average age was 62 years) by verifying if reduction of cIMT in response to therapeutics actually translates into reduction of CVD event risk. They found a positive correlation between interventions that reduce cIMT progression and risk reduction of CVD events, thereby confirming cIMT as a valid surrogate endpoint. The authors suggest that the reason this correlation was observed in clinical trials and not in individual patients is largely due to how clinical trials are set up—i.e., averaging across patients, balancing confounders due to randomization, homogenizing trial cohorts, and constructing higher quality cIMT measurement protocols. They reiterate that cIMT, as measured by ultrasound, displays (a) high correlation with histologically measured vessel wall thickness, (b) evolving reproducibility thanks to clearer measurement standards and technological improvements, (c) close correlation with established CVD, risk factors, atherosclerosis in other vascular beds and occurrence of clinical events, (d) changes over time, and (e) the possibility of being influenced by interventions. Finally, as changes in cIMT due to therapeutic interventions translate into reduced CVD event risk, it represents a credible surrogate endpoint in clinical trials [68].

### 4.3. Carotid Intima Media Thickness in Pediatric Patients with Familial and Polygenic Hypercholesterolemia

A meta-analysis by Narverud et al. that examined markers of atherosclerosis (including cIMT) in children with FH found that cIMT was significantly thicker in FH cohorts than in control groups, though the authors report significant heterogeneity between included studies [46]. The CHARON study examined the effect of 2-year treatment with rosuvastatin on cIMT in children (ages between 6 and 18 years) with heterozygotic FH, and found that, while cIMT was significantly greater relative to their unaffected siblings, treatment resulted in significantly slower progression of cIMT in children with FH, such that after two years the difference in mean cIMT between affected and unaffected children was no longer statistically significant [17]. Similar results were observed in an earlier study comparing the effects of pravastatin and placebo on cIMT measurements in children with FH, where the mean change in CIMT was shown to be significant (and trending towards regression in the treatment arm) [69]. After the study, all patients received pravastatin. In a 10-year follow-up, during which some patients switched to other statins and at the end of which 84% of patients were still using lipid-lowering treatment, cIMT was reassessed and compared with unaffected siblings. The authors found that mean cIMT was greater in patients with FH relative to unaffected siblings, but the progression of cIMT from baseline was similar in both groups. Additionally, age at statin initiation was shown to be significantly associated with cIMT at follow-up [70]. A subsequent 20-year follow-up of the same cohort found that patients with FH had a mean progression of cIMT similar to their unaffected siblings, with 79% of FH patients reporting use of lipid-lowering medication and 84% of those reporting use of prescribed medication in the month before reassessment. The authors suggest that the observed reduction in cIMT progression, combined with no deaths from CVD in the treated cohort, implies an important step towards future cardiovascular risk reduction [30]. It is unlikely that these studies were included in the 2018 meta-analysis by Lorenz et al. [67] or in the 2020 meta-analysis by Willeit et al. [67]. This limits the ability to draw conclusions regarding the suitability of cIMT progression in the analyses. While the data are encouraging, additional randomized control trials with longitudinal follow-ups examining CVD outcomes, safety, and efficacy of therapy, as well as the optimal time for treatment initiation, would be required for more robust conclusions [71].

Patients with PH have been shown to have lower mean cIMT values than FH patients despite comparable LDL-c levels while on treatment, inferring that, while their CVD risk is generally greater than that of the general population, the severity of the disease may be less than in FH patients [72,73]. Though the addition of cIMT to traditional CVD risk prediction models in the normal population leads to only modest improvements, and though genetic analysis is a cornerstone in FH diagnosis, cIMT may prove useful in differentiating FH and PH patients at higher risk from their lower risk counterparts [74]. This notion is further supported by ongoing investigations of new functional parameters (such as three-dimensional cIMT scanning to visualize intima morphology, and cIMT variability to assess surface pattern and extent of intima abnormality), derived from cIMT measurements, which may eventually enable better risk stratification [74]. The most recent European Society of Cardiology/European Atherosclerosis Society guidelines [52] state that cIMT is generally inferior to coronary calcium score in predicting CV events, but add that coronary calcium score is often misleadingly low in patients with FH, even in the homozygous subgroup; therefore, a non-invasive imaging based method for risk stratification in these patients is yet to be elucidated.

## 5. Conclusions

As FH and PH are estimated to be common conditions with potentially severe CVD-related consequences, researchers and clinicians are striving to identify tools to help stratify patients according to their CVD risk, and subsequently optimize treatment. The role of hsCRP in pathogenesis of CVD has not yet been definitively established, and at present it is not recommended as a tool for patient risk stratification. While cIMT appears to be a valid surrogate endpoint in clinical trials, it seems less reliable on an individual basis in adult and pediatric patient populations given a lack of normative values and unified scanning protocols [75]. More methodologically sound research is required to determine whether either parameter may indeed be applied as a tool both in pediatric and adult populations.

## References

[1] Mattiuzzi C., Sanchis-Gomar F., Lippi G. (2019). Worldwide burden of LDL cholesterol: Implications in cardiovascular disease. Nutr. Metab. Cardiovasc. Dis..

[2] Raitakari O.T., Juonala M., Kähönen M., Taittonen L., Laitinen T., Mäki-Torkko N., Järvisalo M.J., Uhari M., Jokinen E., Rönnemaa T. (2003). Cardiovascular risk factors in childhood and carotid artery intima-media thickness in adulthood: The Cardiovascular Risk in Young Finns Study. JAMA.

[3] Zhang Y., Vittinghoff E., Pletcher M.J., Allen N.B., Al Hazzouri A.Z., Yaffe K., Balte P.P., Alonso A., Newman A.B., Ives D.G. (2019). Associations of Blood Pressure and Cholesterol Levels during Young Adulthood with Later Cardiovascular Events. J. Am. Coll. Cardiol..

[4] Talmud P.J., Shah S., Whittall R., Futema M., Howard P., Cooper J.A., Harrison S.C., Li K.W., Drenos F., Karpe F. (2013). Use of low-density lipoprotein cholesterol gene score to distinguish patients with polygenic and monogenic familial hypercholesterolaemia: A case-control study. Lancet.

[5] Nordestgaard B.G., Chapman M.J., Humphries S.E., Ginsberg H.N., Masana L., Descamps O.S., Wiklund O., Hegele R.A., Raal F.J., Defesche J.C. (2013). European Atherosclerosis Society Consensus Panel. Familial hypercholesterolaemia is underdiagnosed and undertreated in the general population: Guidance for clinicians to prevent coronary heart disease: Consensus statement of the European Atherosclerosis Society. Eur. Heart J..

[6] Groselj U., Kovac J., Sustar U., Mlinaric M., Fras Z., Podkrajsek K.T., Battelino T. (2018). Universal screening for familial hypercholesterolemia in children: The Slovenian model and literature review. Atherosclerosis.

[7] Bai B., Yang Y., Wang Q., Li M., Tian C., Liu Y., Aung L.H.H., Li P.-F., Yu T., Chu X.-M. (2020). NLRP3 inflammasome in endothelial dysfunction. Cell Death Dis..

[8] Wiegman A., Gidding S.S., Watts G.F., Chapman M.J., Ginsberg H.N., Cuchel M., Ose L., Averna M., Boileau C., Borén J. (2015). Familial hypercholesterolemia in children and adolescents: Gaining decades of life by optimizing detection and treatment. Eur. Heart J..

[9] Duewell P., Kono H., Rayner K.J., Sirois C.M., Vladimer G., Bauernfeind F.G., Abela G.S., Franchi L., Nuñez G., Schnurr M. (2010). NLRP3 inflammasomes are required for atherogenesis and activated by cholesterol crystals. Nature.

[10] van den Oord S.C., Sijbrands E.J., ten Kate G.L., van Klaveren D., van Domburg R.T., van der Steen A.F.W., Schinkel A.F.L. (2013). Carotid intima-media thickness for cardiovascular risk assessment: Systematic review and meta-analysis. Atherosclerosis.

[11] Lorenz M.W., Markus H.S., Bots M.L., Rosvall M., Sitzer M. (2007). Predicition of Clinical Cardiovascular Events with Carotid Intima-media Thickness. Circulation.

[12] Zimmermann O., Li K., Zaczkiewicz M., Graf M., Liu Z., Torzewski J. (2014). C-Reactive Protein in Human Atherogenesis: Facts and Fiction. Med. Inflamm..

[13] Adukauskienė D., Čiginskienė A., Adukauskaitė A., Pentiokinienė D., Šlapikas R., Čeponienė I. (2016). Clinical relevance of high sensitivity C-reactive protein in cardiology. Medicina.

[14] Rahman T., Hamzan N.S., Mokhsin A., Rahmat R., Ibrahim Z.O., Razali R., Thevarajah M., Nawawi H. (2017). Enhanced status of inflammation and endothelial activation in subjects with familial hypercholesterolaemia and their related unaffected family members: A case control study. Lipids Health Dis..

[15] Calan M., Calan O., Gonen M.S., Bilgir F., Kebapcilar L., Kulac E., Cinali T., Bilgir O. (2011). Examination of adhesion molecules, homocysteine and hs-CRP in patients with polygenic hypercholesterolemia and isolated hypertriglyceridemia. Int. Med..

[16] van’t Klooster C.C., van der Graaf Y., Ridker P.M., Westerink J., Hjortnaes J., Slujis I., Asselbergs F.W., Bots M.L., Kappelle L.J., Visseren F.L.J. (2020). UCC-SMART study group. The relation between healthy lifestlyle changes and decrease in systemic inflammation in patients with stable cardiovascular disease. Atherosclerosis.

[17] Braamskamp M.J.A.M., Langslet G., McCrindle B.W., Cassiman D., Francis G.A., Gagne C., Gaudet D., Morrison K.M., Wiegman A., Turner T. (2017). Effect of Rosuvastatin on Carotid Intima-Media Thickness in Children With Heterozygous Familial Hypercholesterolemia: The CHARON Study (Hypercholesterolemia in Children and Adolescents Taking Rosuvastatin Open Label). Circulation.

[18] Goyal A., Cho L. (2020). Preventive Cardiology and Risk Assessment: Beyond LDL. Curr. Atheroscler. Rep..

[19] Hu P., Dharmayat K.I., Stevens C.A.T., Sharabiani M.T.A., Jones R.S., Watts G.F., Genest J., Ray K.K., Vallejo-Vaz A.J. (2020). Prevalence of Familial Hypercholesterolemia among the General Population and Patients with Atherosclerotic Cardiovascular Disease: A Systematic Review and Meta-Analysis. Circulation.

[20] Sharifi M., Futema M., Nair D., Humphries S.E. (2017). Genetic Architecture of Familial Hypercholesterolaemia. Curr. Cardiol. Rep..

[21] Hajighasemi S., Mahdavi Gorabi A., Bianconi V., Pirro M., Banach M., Tafti H.A., Reiner Ž., Sahebkar A. (2019). A review of gene- and cell-based therapies for familial hypercholesterolemia. Pharmacol. Res..

[22] Tada H., Kawashiri M.A., Nohara A., Inazu A., Kobayashi J., Mabuchi H., Yamigishi M. (2014). Autosomal Recessive Hypercholesterolemia: A Mild Phenotype of Familial Hypercholesterolemia: Insight from the Kinetic Study using Stable Isotope and Animal Studies. J. Atheroscler. Thromb..

[23] Futema M., Bourbon M., Williams M., Humphries S.E. (2018). Clinical utility of the polygenic LDL-C SNP score in familial hypercholesterolemia. Atherosclerosis.

[24] Wang J., Dron J.S., Ban M.R., Robinson J.F., McIntyre A.D., Alazzam M., Zhao P.J., Dilliott A.A., Cao H., Huff M.W. (2016). Polygenic Versus Monogenic Causes of Hypercholesterolemia Ascertained Clinically. Arterioscler. Thromb. Vasc. Biol..

[25] Singh S., Bittner V. (2015). Familial Hypercholesterolemia—Epidemiology, Diagnosis, and Screening. Curr. Atheroscler. Rep..

[26] Cuchel M., Bruckert E., Ginsberg H.N., Raal F.J., Santos R.D., Hegele R.A., Kuivenhoven J.A., Nordestgaard B.G., Descamps O.S., Steinhagen-Thiessen E. (2014). European Atherosclerosis Society Consensus Panel on Familial Hypercholesterolaemia. Homozygous familial hypercholesterolaemia: New insights and guidance for clinicians to improve detection and clinical management. A position paper from the Consensus Panel on Familial Hypercholesterolaemia of the European Atherosclerosis Society. Eur. Heart J..

[27] Dron J.S., Hegele R.A. (2018). Polygenic influences on dyslipidemias. Curr. Opin. Lipidol..

[28] Martin A.C., Gidding S.S., Wiegman A., Watts G.F. (2017). Knowns and unknowns in the care of pediatric hypercholesterolemia. J. Lipid Res..

[29] Vuorio A., Kuoppala J., Kovanen P.T., Humphries S.E., Tonstad S., Wiegman A., Drogari E., Ramaswami U. (2017). Statins for children with familial hypercholesterolemia. Cochrane Database Syst. Rev..

[30] Luirink I.K., Wiegman A., Kusters D.M., Hof M.H., Groothoff J.W., de Groot E., Kastelein J.J.P., Hutten B.A. (2019). 20-Year Follow-up of Statins in Children with Familial Hypercholesterolemia. N. Engl. J. Med..

[31] Yousuf O., Mohanty B.D., Martin S.S., Joshi P.H., Blaha M.J., Nasir K., Blumenthal R.S., Budoff M.J. (2013). High-sensitivity C-reactive protein and cardiovascular disease: A resolute belief or an elusive link?. J. Am. Coll. Cardiol..

[32] van den Berg C.W., Taylor K.E., Lang D. (2004). C-Reactive Protein-Induced In Vitro Vasorelaxation Is an Artefact Caused by the Presence of Sodium Azide in Commercial Preparations. Arterioscler. Thromb. Vasc. Biol..

[33] Taylor K.E., Giddings J.C., van den Berg C.W. (2005). C-Reactive Protein-Induced In Vitro Endothelial Cell Activation Is an Artefact Caused by Azide and Lipopolysaccharide. Arterioscler. Thromb. Vasc. Biol..

[34] Devaraj S., Yun J.-M., Adamson G., Galvez J., Jialal I. (2009). C-reactive protein impairs the endothelial glycocalyx resulting in endothelial dysfunction. Cardiovasc. Res..

[35] Bisoendial R.J., Kastelein J.J., Levels J.H., Zwaginga J.J., van den Bogaard B., Reitsma P.H., Meijers J.C.M., Hartman D., Levi M., Stroes E.S.G. (2005). Activation of inflammation and coagulation after infusion of C-reactive protein in humans. Circ. Res..

[36] Bisoendial R., Kastelein J., Stroes E. (2005). In response to van den Berg et al.: On the direct actions of CRP in humans. Circ. Res..

[37] Koenig W. (2013). High-sensitivity C-reactive protein and atherosclerotic disease: From improved risk prediction to risk-guided therapy. Int. J. Cardiol..

[38] Ridker P.M., Glynn R.J., Hennekens C.H. (1998). C-reactive protein adds to the predictive value of total and HDL cholesterol in determining risk of first myocardial infarction. Circulation.

[39] Ridker P.M., Hennekens C.H., Buring J.E., Rifai N. (2000). C-reactive protein and other markers of inflammation in the prediction of cardiovascular disease in women. N. Engl. J. Med..

[40] Kaptoge S., Di Angelantonio E., Lowe G., Pepys M.B., Thompson S.G., Collins R., Danesh J., Emerging Risk Factors Collaboration (2010). C-reactive protein concentration and risk of coronary heart disease, stroke, and mortality: An individual participant meta-analysis. Lancet.

[41] Emberson J., Bennett D., Link E., Parish S., Danesh J., Armitage J., Collins R., Heart Protection Study Collaborative Group (2011). C-reactive protein concentration and the vascular benefits of statin therapy: An analysis of 20,536 patients in the Heart Protection Study. Lancet.

[42] Shah T., Casas J.P., Cooper J.A., Tzoulaki I., Sofat R., McCormack V., Smeeth L., Deanfield J.E., Lowe G.D., Rumley A. (2009). Critical appraisal of CRP measurement for the prediction of coronary heart disease events: New data and systematic review of 31 prospective cohorts. Int. J. Epidemiol..

[43] Schnell-Inderst P., Schwarzer R., Göhler A., Grandi N., Grabein K., Stollenwerk B., Manne J., Klauss V., Siebert U., Wasem J. (2010). Prognostic value, clinical effectiveness, and cost-effectiveness of high-sensitivity C-reactive protein as a marker for major cardiac events in asymptomatic individuals: A health technology assessment report. Int. J. Technol. Assess. Health Care.

[44] Piepoli M.F., Hoes A.W., Agewall S., Albus C., Brotons C., Catapano A.L., Cooney M.-T., Corrà U., Cosyns B., Deaton C. (2016). 2016 European Guidelines on cardiovascular disease prevention in clinical practice: The Sixth Joint Task Force of the European Society of Cardiology and Other Societies on Cardiovascular Disease Prevention in Clinical Practice (constituted by representatives of 10 societies and by invited experts) Developed with the special contribution of the European Association for Cardiovascular Prevention & Rehabilitation (EACPR). Eur. Heart J..

[45] van Wissen S., Trip M.D., Smilde T.J., de Graaf J., Stalenhoef A.F., Kastelein J.J. (2002). Differential hs-CRP reduction in patients with familial hypercholesterolemia treated with aggressive or conventional statin therapy. Atherosclerosis.

[46] Narverud I., Retterstøl K., Iversen P.O., Halvorsen B., Ueland T., Ulven S.M., Ose L., Aukrust P., Veierød M.B., Holven K.B. (2014). Markers of atherosclerotic development in children with familial hypercholesterolemia: A literature review. Atherosclerosis.

[47] Narverud I., van Lennep J.R., Christensen J.J., Versmissen J., Gran J.M., Iversen P.O., Aukrust P., Halvorsen B., Ueland T., Ulven S.M. (2015). Maternal inheritance does not predict cholesterol levels in children with familial hypercholesterolemia. Atherosclerosis.

[48] Ueland T., Vissers M.N., Wiegman A., Rodenburg J., Hutten B., Gullestad L., Ose L., Rifai N., Ridker P.M., Kastelein J.J.P. (2006). Increased inflammatory markers in children with familial hypercholesterolaemia. Eur. J. Clin. Investig..

[49] Guran O., Akalin F., Ayabakan C., Dereli F.Y., Haklar G. (2007). High-sensitivity C-reactive protein in children at risk for coronary artery disease. Acta Paediatr..

[50] Martino F., Carlomosti F., Avitabile D., Persico L., Picozza M., Barillà F., Arca M., Montali A., Martino E., Zanoni C. (2015). Circulating miR-33a and miR-33b are up-regulated in familial hypercholesterolaemia in paediatric age. Clin. Sci..

[51] Christensen J.J., Osnes L.T., Halvorsen B., Retterstøl K., Bogsrud M.P., Wium C., Svilaas A., Narverud I., Ulven S.M., Aukrust P. (2017). Altered leukocyte distribution under hypercholesterolemia: A cross-sectional study in children with familial hypercholesterolemia. Atherosclerosis.

[52] Mach F., Baigent C., Catapano A.L., Koskinas K.C., Casula M., Badimon L., Chapman M.J., De Backer G.G., Delgado V., Ference B.A. (2020). 2019 ESC/EAS Guidelines for the management of dyslipidaemias: Lipid modification to reduce cardiovascular risk. Eur. Heart J..

[53] Touboul P.J., Hennerici M.G., Meairs S., Adams H., Amarenco P., Bornstein N., Csiba L., Desvarieux M., Ebrahim S., Hernandez R.H. (2012). Mannheim carotid intima-media thickness and plaque consensus (2004-2006-2011). An update on behalf of the advisory board of the 3rd, 4th and 5th watching the risk symposia, at the 13th, 15th and 20th European Stroke Conferences, Mannheim, Germany, 2004, Brussels, Belgium, 2006, and Hamburg, Germany, 2011. Cerebrovasc. Dis..

[54] Pignoli P., Tremoli E., Poli A., Oreste P., Paoletti R. (1986). Intimal plus medial thickness of the arterial wall: A direct measurement with ultrasound imaging. Circulation.

[55] Liao X., Norata G.D., Polak J.F., Steuhower C.D.A.A., Catapano A., Rundek T., Ezhov M., Sander D., Thompson S.G., Lorenz M.W. (2015). Normative values for carotid intima media thickness and its progression: Are they transferrable outside of their cohort of origin?. Eur. J. Prev. Cardiol..

[56] Lorenz M.W., Polak J.F., Kavousi M., Mathiesen E.B., Völzke H., Tuomainen T.-P., Sander D., Plichart M., Catapano A.L., Robertson C.M. (2012). Carotid intima-media thickness progression to predict cardiovascular events in the general population (the PROG-IMT collaborative project): A meta-analysis of individual participant data. Lancet.

[57] Engelen L., Ferreira I., Stehouwer C.D., Boutouyrie P., Laurent S. (2013). Reference Values for Arterial Measurements Collaboration. Reference intervals for common carotid intma-media thickness measured with echotracking: Relation with risk factors. Eur. Heart J..

[58] Gooty V.D., Sinaiko A.R., Ryder J.R., Dengel D.R., Jacobs D.R., Steinberger J. (2018). Association Between Carotid Intima Media Thickness, Age, and Cardiovascular Risk Factors in Children and Adolescents. Metab. Syndr. Relat. Disord..

[59] Baroncini L.A., Sylvestre Lde C., Pecoits Filho R. (2016). Assessment of Intima-media Thickness in Healthy Children Aged 1 to 15 Years. Arq. Bras. Cardiol..

[60] Zanini J.L.S.S., Rodrigues T.M.B., Barra C.B., Filgueiras M.F.T.F., Silva I.N. (2019). Intima-Media Thickness of the Carotid Arteries is Affected by Pubertal Maturation in Healthy Adolescents. Rev. Paul. Pediatr..

[61] Calabrò M.P., Carerj S., Russo M.S., Salvatore M., De Luca F.L., Onofrio M.T.N., Antonini-Canterin F., Zito C., Oreto L., Manuri L. (2017). Carotid artery intima-media thickness and stiffness index β changes in normal children: Role of age, height and sex. J. Cardiovasc. Med..

[62] Curcio S., García-Espinosa V., Arana M., Farro I., Chiesa P., Giachetto G., Zócalo Y., Bia D. (2016). Growing-Related Changes in Arterial Properties of Healthy Childre, Adolescents and Adults Nonexposed to Cardiovascular Risk Factors: Analysis of Gender-Related Differences. Int. J. Hypertens..

[63] Koçyiğit A., Doğan M., Yilmaz İ., Çağlar M., Hatipoğlu C., Koçyiğit F., Herek D., Karabulut N. (2014). Relation of age and sex with carotid intima media thickness in healthy children. Turk. J. Med. Sci..

[64] Urbina E.M., Williams R.V., Alpert B.S., Collins R.T., Daniels S.R., Hayman L., Jacobson M., Mahoney L., Mietus-Snyder M., Rocchini A. (2009). Noninvasive Assessment of Subclinical Atherosclerosis in Children and Adolescents: Recommendations for Standard Assessment for Clinical Research: A Scientific Statement from the American Heart Association. Hypertension.

[65] Dalla Pozza R., Ehringer-Schetitska D., Fritsch P., Jokinen E., Petropoulos A., Oberhoffer R., Association for European Pediatric Cardiology Working Group Cardiovascular Prevention (2015). Intima media thickness measurement in children: A statement from the Association for European Paediatric Cardiology (AEPC) Working Group on Cardiovascular Prevention endorsed by the Association for European Paediatric Cardiology. Atherosclerosis.

[66] Polak J.F., Pencina M.J., O’Leary D.H., D’Agostino R.B. (2011). Common carotid artery intima-media thickness progression as a predictor of stroke in multi-ethnic study of atherosclerosis. Stroke.

[67] Lorenz M.W., Gao L., Ziegelbauer K., Norata G.D., Empana J.P., Schmidtmann I., Lin H.-J., McLachlan S., Bokemark L., Ronkainen K. (2018). Predictive value for cardiovascular events of common carotid intima media thicknes and its rate of change in individuals at high cardiovascular risk—Results from the PROG-IMT collaboration. PLoS ONE.

[68] Willeit P., Tschiderer L., Allara E., Reuber K., Seekircher L., Gao L., Liao X., Lonn E., Gerstein H.C., Yusuf S. (2020). Carotid Intima-Media Thickness Progression as Surrogate Marker for Cardiovascular Risk: Meta-Analysis of 119 Clinical Trials Involving 100,667 Patients. Circulation.

[69] Wiegman A., Hutten B.A., de Groot E., Rodenburg J., Bakker H.D., Büller H.R., Sijgbrands E.J.G., Kastelein J.J.P. (2004). Efficacy and safety of statin therapy in children with familial hypercholesterolemia: A randomized controlled trial. J. Am. Med. Assoc..

[70] Kusters D.M., Avis H.J., de Groot E., Wijburg F.A., Kastelein J.J.P., Wiegman A., Hutten B.A. (2014). Ten-Year Follow-up After Initiation of Statin Therapy in Children with Familial Hypercholesterolemia. J. Am. Med. Assoc..

[71] Maliachova O., Stabouli S. (2018). Familial Hypercholesterolemia in Children and Adolescents: Diagnosis and Treatment. Curr. Pharm. Des..

[72] Sharifi M., Higginson E., Bos S., Gallivan A., Harvey D., Li K.W., Abeysekera A., Haddon A., Ashby H., Shipman K.E. (2017). Greater preclinical atherosclerosis in treated monogenic familial hypercholesterolemia vs. polygenic hypercholesterolemia. Atherosclerosis.

[73] Sharifi M., Futema M., Nair D., Humphries S.E. (2019). Polygenic Hypercholesterolemia and Cardiovascular Disease Risk. Curr. Cardiol. Rep..

[74] Sharifi M., Rakhit R.D., Humphries S.E., Nair D. (2016). Cardiovascular risk stratification in familial hypercholesterolemia. Heart.

[75] Drole Torkar A., Plesnik E., Groselj U., Battelino T., Kotnik P. (2020). Carotid Intima-Media Thickness in Healthy Children and Adolescents: Normative Data and Systematic Literature Review. Front. Cardiovasc. Med..

